# Implementation of an NGS panel for clinical practice in paraffin-embedded tissue samples from locally advanced and metastatic melanoma patients

**DOI:** 10.37349/etat.2020.00006

**Published:** 2020-04-28

**Authors:** Paola Castillo, Marta Marginet, Pedro Jares, Mireia García, Elena Gonzalvo, Ana Arance, Adriana García, Llucia Alos, Cristina Teixido

**Affiliations:** 1 Department of Pathology, Hospital Clinic, IDIBAPS, University of Barcelona, 08036 Barcelona, Spain; 2 Department of Medical Oncology, Hospital Clinic, IDIBAPS, University of Barcelona, 08036 Barcelona, Spain; University of Southampton, UK

**Keywords:** Melanoma, Idylla, real-time polymerase chain reaction, BRAF, next-generation sequencing

## Abstract

**Aim:**

Single biomarker diagnostic test of *BRAF*V600 locus in metastatic melanoma is mandatory for treatment decision; however, multiple-gene based techniques, such as targeted next-generation sequencing (NGS) are being used to maximize the number of patients that can benefit from a targeted therapy. The main objective of this study is to investigate whether an NGS panel could be adopted in routine clinical care for advanced melanoma.

**Methods:**

Patients diagnosed with advanced melanoma at our center from 2017 to 2019 were included. Presence of genetic alterations was performed using two methods: real-time polymerase chain reaction-based Idylla test (Biocartis) and NGS with the oncomine solid tumor DNA kit (Thermo Fisher Scientific). Total genomic DNA was extracted from formalin-fixed and paraffin embedded samples for sequencing.

**Results:**

A total of 155 samples were evaluated for molecular analysis but 40 samples (25.8%) were inadequate for sequencing. The clinical utility of *BRAF*V600 real-time polymerase chain reaction and targeted-NGS was compared in 29 samples and a very good concordance was observed (Kappa = 0.89, 95% confidence interval 0.68 ± 1.05). An oncogenic mutation by NGS was found in 75 samples (65%)–53% of whom were candidates for personalized therapies. The most prevalent mutated genes were *BRAF* (39%), *TP53* (23%), and *NRAS* (14%). Other genes identified at lower incidence (< 5%) were: *PIK3CA*, *ERBB4*, *CTNNB1*, *STK11*, *FGFR1*, *SMAD4*, *KRAS*, *FGFR3*, *PTEN* and *AKT*. Co-occurrence of oncogenic mutations was detected in 40% of the samples. Among the mutations identified, *TP53* was significantly more prevalent in men (men 31.8% *versus* women 12.2%, *P* = 0.03) and *NRAS* in women (men 9.1% *versus* women 24.4%, *P* = 0.03).

**Conclusions:**

Targeted-NGS testing is a feasible technique to implement in the routine clinical practice. Based on our results, NGS has provided more information on target-genes than RT-PCR technique, maximizing the benefit for patients with advanced melanoma.

## Introduction

In the last decade, the treatment of oncology patients has made a major shift from a one-size-fits-all approach towards personalized medicine. The use of genomic diagnostic technologies integrated in the clinical setting is a key tool for patients-tailored treatments [1, 2]. In widespread melanoma disease, treatments have been historically palliative and mostly ineffective. The identification of oncogenic mutations as drivers in advanced disease provide targeted therapies that have led to improvements in response rates and survival in a subgroup of patients [3–5]. The most relevant mutated gene is *BRAF*, which is mutated in about 50% to 70% of cutaneous melanomas, and the most common genetic change is a glutamic acid for valine substitution at position 600 (V600E) [6–9]. *BRAF* mutations may be classified based on their mechanisms to activate the mitogen activated protein kinase (MAPK) pathway into three categories [10, 11]: *BRAF* class I mutations with kinase activity involving codon 600; class II mutations, with high or intermediate *BRAF* kinase activity involving codons outside V600 locus and class III mutations, with no or low *BRAF* kinase activity. Mainly, *BRAF* class I mutations are targetable alterations with approved therapies [12]. Consequently, *BRAF* testing is considered mandatory in patients with advanced (unresectable or metastatic) melanomas stage III or stage IV, and is highly recommended in high-risk resected disease stage IIC [13]. Traditionally, these analyzes have been based on the isolated *BRAF*V600E identification using real-time quantitative polymerase chain reaction (RT-PCR), but clinically validated next-generation sequencing (NGS) panels covering key oncogenic drivers are increasingly being performed routinely. Moreover, when the result of a case is negative for class I *BRAF* alterations, it is recommended to sequence the other *BRAF* loci (class II and III alterations) to confirm the wild-type (wt) status, as well as sequencing *NRAS* and *c-kit* genes, in order to stratify patients in clinical trials [11, 13]. Thus, as therapies for locally advanced and metastatic melanoma become more complex, and more targetable drivers’ alterations modulating the clinical decisions are found, there is an urgent need to validate and establish diagnostic algorithms for molecular testing at every institution according to established guidelines [14–16].

To achieve a more complete molecular diagnosis, the main objective of the present study was to evaluate the implementation of the diagnostic oncomine solid tumor (OST) NGS panel, based on the ion torrent sequencing technology, in formalin-fixed paraffin-embedded (FFPE) tissue samples to replace the current RT-PCR routine diagnostic tool for *BRAF*V600 locus test. Thus, we performed a comparison of both approaches (RT-PCR and NGS) for *BRAF*V600 status. Additionally, we described the spectrum of somatic mutations identified by NGS in a series of advanced melanoma samples in our clinical setting.

## Materials and methods

### Clinical samples

Between June 2017 and October 2019, a total of 155 FFPE melanoma samples were submitted for molecular studies with prior full informed consent of the patients and approval from the Internal Review Board of the Hospital Clinic of Barcelona (Barcelona, Spain). Metastatic melanoma samples were prioritized for molecular studies. However, when this material was insufficient or inadequate for molecular analysis (< 20% of tumor cells), the primary melanoma samples were assessed. The demographic data were retrieved from the electronic medical records of Hospital Clinic of Barcelona. All test results were documented in the patient’s files.

Before any test was performed, the tumor content of tissue samples was evaluated by estimating the percentage of neoplastic cells on hematoxylin and eosin-stained whole slides. The percentage of neoplastic cells in the samples ranged from 10% to 95%.

### Idylla BRAF mutation test

The Idylla test (Biocartis, Mechelen, Belgium) is a fully automated RT-PCR system that consists of three allele-specific PCR reactions that enable identification of *BRAF* wt, *BRAF*V600E/E2/D, or *BRAF*V600K/R/M sequence [17]. For analyses, two 10 μm thick tissue sections were transferred into the cartridge and performed according to manufacturer’s recommendations.

### DNA isolation

Five 10 μm thick tissue sections were used for DNA extraction using the QIAamp DNA FFPE Tissue Kit (Qia-gen, Hilden, Germany), according to manufacturer’s instructions. DNA content and quality were determined using the Qubit dsDNA HS assay (Life Technologies, Gaithersburg, USA).

### Targeted NGS

An input of 10 ng of DNA was used as a template to generate libraries using the OST DNA kit (Thermo Fisher Scientific, Massachusetts, USA) following the manufacturer’s instructions. The panel can identify somatic mutations (substitutions, insertions, deletions and inversions) in the following 22 genes: *EGFR*, *ALK*, *ERBB2*, *ERBB4*, *FGFR1*, *FGFR2*, *FGFR3*, *MET*, *DDR2*, *KRAS*, *PIK3CA*, *BRAF*, *AKT1*, *PTEN*, *NRAS*, *MAP2K1*, *STK11*, *NOTCH1*, *CTNNB1*, *SMAD4*, *FBXW7* and *TP53*. Sequencing was performed using the Ion Personal Genome Machine (Thermo Fisher Scientific) platform and analyzed by Ion Reporter Server (Thermo Fisher Scientific) according to the manufacturer’s instructions.

### Statistical analysis

Concordance between the Idylla *BRAF* mutation test and the NGS OST DNA kit for *BRAF*V600 mutation identification was evaluated using the kappa statistic and interpreted as suggested by Landis and Koch [18]. Data were analyzed with the SPSS statistical package (version 23.0; SPSS Inc., Chicago, IL, USA). The test used for comparison of qualitative variables was chi-square test. Significance was considered with an alpha risk of 0.05.

## Results

### Patients’ demographics and samples

A total of 155 FFPE tumor samples from 147 patients with locally advanced or metastatic melanoma were prospectively tested to identify therapeutic options. The tumor specimens were dated from 2006 to 2019. All samples were derived from either excision of primary melanoma (*N* = 84) and from biopsies or resections of metastasis (*N* = 71). Patients’ median age was 65 years (interquartile range of 10–89 years). Ninety out of 147 patients (61.2%) were male and 57 (38.8%) were female. Most patients had non-acral cutaneous melanoma (*N* = 125, 85%), followed by mucosal melanoma (*N* = 9, 6.1%), acral lentiginous melanoma (*N* = 8, 5.4%) and uveal melanoma (*N* = 1, 0.7%). Four patients were diagnosed with melanoma of unknown primary (2.7%).

**Figure 1.**
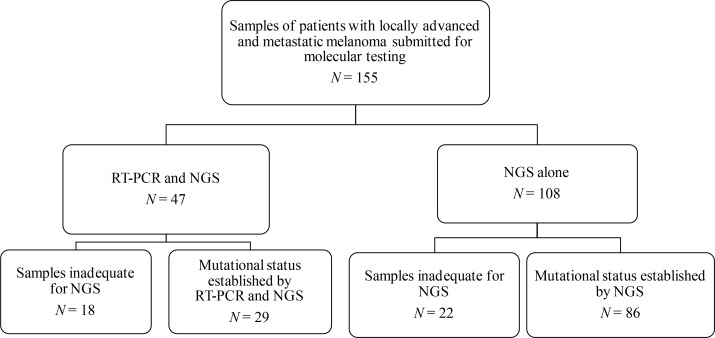
Flow chart of the patient cohort

Two different techniques were compared to validate and to implement the NGS technique in our routine clinical practice for melanoma patients (Figure 1).

### Concordance between RT-PCR and NGS techniques for *BRAF* mutation

RT-PCR was the standard routine diagnostic tool for *BRAF*V600 mutation analysis in advanced melanoma patients at our institution. In order to directly compare clinical utility of RT-PCR and NGS techniques for *BRAF* testing, we further performed an NGS panel in parallel in those patients with additional tumor material and determined the concordance between NGS and RT-PCR data of *BRAF* status as the reference. Eighteen samples of the 47 cases intended to be included in the comparison study, were inadequate for NGS analysis (38.3%) (Figure 1). Fifteen of the inadequate samples did not have enough tumor material in the FFPE block and three had insufficient DNA quantity or quality for sequencing. *BRAF*V600 status was established in all samples by RT-PCR (47/47, 100%). Eighteen of these cases were positive for *BRAF*V600E/E2/D mutation (18/47, 38.3%). NGS was successfully performed in 29 cases (29/47, 61.7%) and 14 samples were considered positive for *BRAF* (48.3%). Table 1 shows the concordance on *BRAF* mutational status between RT-PCR and NGS methods.

**Table 1.** Concordance of BRAF mutational status established by RT-PCR and NGS methods

		**RT-PCR**		Total
		***BRAF* mutated**	***BRAF* wild-type**	
NGS	*BRAF* mutated	**12**	2	14
	*BRAF* wild-type	0	**15**	15
	Total	12	17	29

Kappa = 0.89, 95% confidence interval (CI) 0.68 ± 1.05. Concordance samples are in bold.

A very good concordance among the two tested techniques was observed for the characterization of patients according to *BRAF* mutation (Kappa = 0.89, 95% CI 0.68 ± 1.05). Discordance was identified only in two samples. Two *BRAF* alterations were detected by NGS but missed by RT-PCR (*BRAF*L597S, allele frequency 73% and *BRAF*V600K, allele frequency 31%). Among them, *BRAF*V600K but not *BRAF*L597S was covered by the RT-PCR test used. On the other hand, the sample identified by NGS but not by RT-PCR, a *BRAF* mutation in codon V600K, measured 100 mm^2^ with a tumor representation of 80% and a DNA concentration of 87.1 ng/μl.

### Somatic mutations detected by NGS

Out of the 155 samples collected, a total of 115 (74.2%) yielded an informative DNA sequencing result. As commented in methods, all samples processed by NGS contained at least 20% of tumor cells (mean percentage of tumor cells of 80%). NGS analysis failed to deliver a mutational status (inadequate samples) when a previous molecular determination was performed (RT-PCR) in comparison to molecularly undetermined samples (18/47, 38% *versus* 22/108, 20%) (Figure 1). RT-PCR was prioritized when the tumor tissue was insufficient to perform both techniques in the comparison study. In the majority of cases, inadequate samples corresponded to FFPE blocks without enough tumor material (RT-PCR and NGS 15/18, 83%; NGS alone 16/22, 73%) (Figure 1). The other nine samples not suitable for NGS analysis were due to lack of a sufficient amount of DNA or insufficient quality for genomic DNA sequencing.

**Figure 2.**
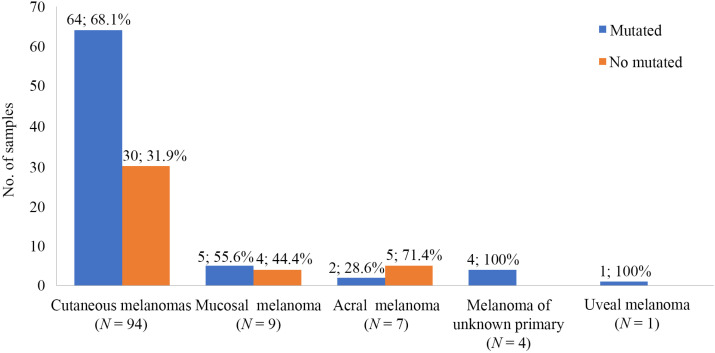
Melanoma subtypes distribution of the melanoma samples successfully genotyped by NGS (*N* = 115)

The melanoma types of all tumors successfully genotyped in the series are shown in Figure 2. The majority of the molecularly characterized melanomas were cutaneous type melanomas (81.7%, 94/115). In this subtype, the incidence of alterations identified in our cohort was 68.1% (64/94), whereas the percentage of cases with a mutation reported in mucosal and acral melanomas was lower, 55.6% (5/9) and 28.6% (2/7), respectively.

A somatic alteration was found in 65.2% of the tumor tissue samples tested (75/115). NGS identified a total of 114 somatic mutations, an average of 0.99 alterations per sample. The genes with the highest frequency were *BRAF* (39%, 45/114), *TP53* (23%, 26/114), *NRAS* (14%, 16/114), *PIK3CA* (4%, 5/114) and *ERBB4* (3%, 4/114). Other genes identified with lower incidence (< 3%) were: *CTNNB1*, *STK11*, *FGFR1*, *SMAD4*, *KRAS*, *FGFR3*, *PTEN* and *AKT1*.

**Figure 3.**
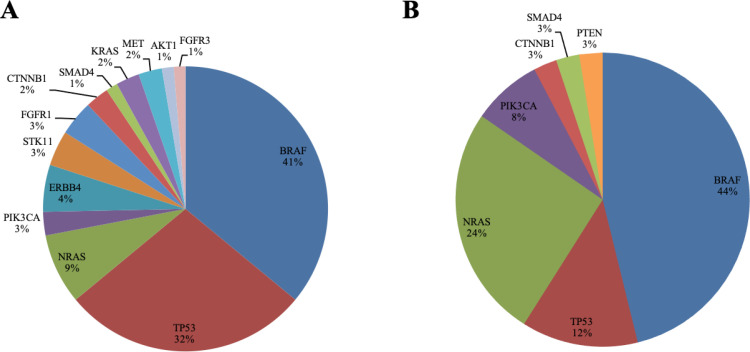
Pie charts representing the frequencies (%) of the examined genes identified segregated by gender; men (A) and women (B) from 107 patients included in the cohort. Frequencies are expressed as the percentage of positive samples for each molecular alteration relative to the total number of patients with an informative molecular result

Frequencies of the altered genes according to gender were evaluated (Figure 3A and 3B). Molecular alterations were more commonly found in men (69.7%, 46/66) than in women (63.4%, 26/41). Melanomas from female patients tended to yield more oncogenic mutations by a single gene than from males (74.1% *versus* 51.1%, *P* = 0.09). Furthermore, a higher diversity of mutations and a higher number of alterations per sample were observed in the cohort of men (men 75/66 *versus* women 39/41). Mutations in *ERBB4*, *STK11*, *FGFR1*, *FGFR3*, *KRAS*, *MET* and *AKT1* genes were only present in men, while the only *PTEN* alteration was found in a sample from a female patient. No significant differences were discerned in the prevalence of *BRAF* (men 27/66, 40.9% *versus* women 18/41, 43.9%), but *TP53* was significantly more prevalent in men (men 21/66, 31.8% *versus* women 5/41, 12.2%, *P* = 0.03), while NRAS was prevalent in women (men 6/66, 9.1% *versus* women 10/41, 24.4%, *P* = 0.03).

**Figure 4.**
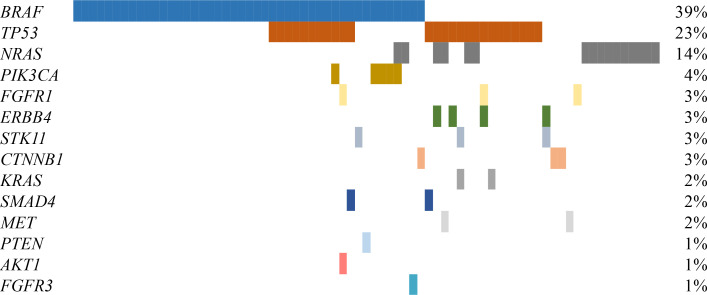
Spectrum of genes with alterations identified in 115 advanced melanomas. The percentage of samples with an alteration detected is noted at the right. Samples with an alteration identified are displayed as columns (*N* = 75)

Among patients with at least one molecular alteration identified, co-occurrence of mutations was detected in 40% of cases (30/75) (Figure 4). Of those, 22 cases presented two alterations, seven presented three, and one patient was reported with four different mutations. Alterations of *TP53* were concomitant in 76.9% of cases (20/26). *BRAF* was identified co-mutated in 44% of cases (20/45), *TP53* being the most common concomitant alteration identified (55%, 11/20). All the *PIK3CA* (*N* = 5), *FGFR3* (*N* = 1), *PTEN* (*N* = 1) and *AKT1* (*N* = 1) alterations identified were concomitant with *BRAF* mutations. On the other hand, *NRAS* alterations were more commonly found as single mutations (62%, 10/16). Within these cases, two were co-mutated with *BRAF*. Moreover, one of the two identified *KRAS* alterations presented a concomitant mutation with *STK11*.

### BRAF mutation types identified by NGS

**Figure 5.**
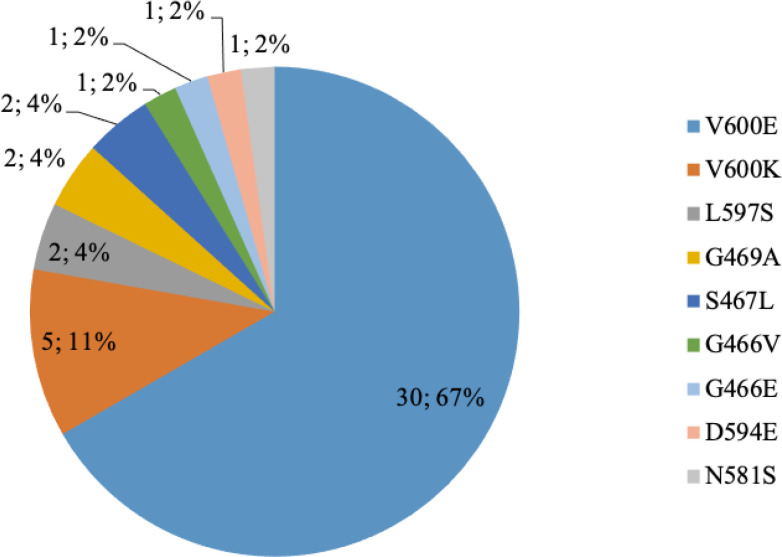
Schematic representation of the distribution of the different *BRAF* mutations (*N* = 45) identified in 115 samples from advanced melanomas by NGS

*BRAF* was the most prevalent mutated gene in our cohort (39%, 45/115) and was identified throughout all melanoma site distribution except in uveal melanoma. All melanoma of unknown primary origin harbored a *BRAF* alteration (100%, 4/4 samples), whereas an alteration of *BRAF* gene was recorded in 40.4 % (38/94) of cutaneous melanomas, 28.6% (2/7) of acral melanomas and in 11.1% (1/9) of the mucosal melanomas. Heterogeneity and frequency of *BRAF* mutations are shown in Figure 5. *BRAF* mutations mainly affected codon 600 of the *BRAF* gene (77.8%, 35/45), *BRAF*V600E being the most common mutation found (67%, 30/45). In detail, a total of nine different mutations were identified, namely: p.Val600Glu (V600E), p.Val600Lys (V600K), p.Leu597Ser (L597S), p.Gly469Ala (G469A), p.Ser467Leu (S467L), p.Gly466Val (G466V), p.Gly466Glu (G466E), p.Asp594Glu (D594E), p.Asn581Ser (N581S).

## Discussion

As personalized medicine is constantly evolving, the identification and implementation of techniques to identify new actionable genes is of great importance. Integrated genomic diagnostic technologies, such as NGS gene panels have revolutionized clinical genomics and emerged as key drivers of personalized cancer care and cancer discovery, because they can simultaneously screen for multiple genes in a single assay optimizing tumor tissue requirements. NGS gene panels can be commercial or custom designed, depending on the specific gene targeting needs, although a validation is needed in both scenarios [19, 20]. Here, we have presented valuable real data on the implementation of a commercial and validated test with European conformity *in vitro* diagnostic for multiple molecular-targeted therapies, including *BRAF*, in a Spanish cohort of advanced melanoma. To this end, we have compared the Idylla BRAF test (RT-PCR), a proven robust and reliable technique for the characterization of *BRAF* [21, 22], against the OST DNA kit of 22 genes (NGS) in a subset of samples. To the best of our knowledge, this is the first prospective NGS study and the largest in a Spanish cohort of advanced melanoma patients. In addition, the present study has used a commercial NGS with a panel of pan-cancer genes to optimize institutional resources and increase profitability by implementing it in different pathologies, including melanoma.

In our series, the most common location of melanoma was the skin, as it has been described in other Western countries and Japan [23, 24]. We observed the expected frequency of described *BRAF* mutated melanomas (38%) in the subset of samples where the technique was performed [3, 25, 26]. Furthermore, we obtained a good concordance between RT-PCR and NGS techniques (Kappa = 0.89, 95% CI 0.68 ± 1.05) in agreement with other studies [20–22, 27, 28]. Only two discrepancies were found in the comparison study (*BRAF*V600K and *BRAF*L597S), but *BRAF*L597S mutation was not detected by RT-PCR because it was not covered by the test used. *BRAF* discrepancies between Cobas (RT-PCR) and NGS techniques have also been reported in selected cases, as in the study by Reiman et al. [20].

Despite the good concordance obtained between RT-PCR and NGS, the turnaround time and cost of the NGS as compared to the RT-PCR test, might lower its applicability for daily diagnosis for some institutions given that it requires experience in the wet lab, specific platforms, bioinformatics and it is a more complex assay. The simplicity of the technology chosen is being considered in busy routine laboratories and many still prefer the solution of PCR techniques. Nonetheless, de Unamuno Bustos et al. [29], demonstrated the cost-effectiveness and lower turn-around-time for NGS-based analysis compared to conventional methods in melanoma samples. Our results provide further information on the great benefits of using NGS tests. The OST panel in our study found 75 mutated samples (65%) and delivered additional information beyond mutations in *BRAF*V600 locus. *BRAF* class I mutations, consistent with published literature, were the most common mutation identified (39%) and the most predominant among cutaneous melanomas, but not the only class mutation type identified [8, 26, 30]. Guidelines for negative cases of *BRAF* class I alterations recommend sequencing the loci of the other known less frequent *BRAF* mutations (class II and class III *BRAF* alterations) to confirm *BRAF* wt status, as well as to test these cases for *NRAS* and *c-kit* mutations. Unlike RT-PCR, NGS enables testing of these and other alterations in once without performing further tests and reducing the final turnaround time. The main weakness of our panel is the absence of *c-kit* gene, reported in mucosal and acral melanomas. However, these are infrequent types of melanoma and represent 11% of the cases included in our series.

NGS allowed identification of *BRAF* class II alterations, as well as *NRAS* mutations, which may be relevant for ongoing or future clinical trials. *NRAS* mutation, described as the second most common oncogenic alteration in melanoma (20%), was associated with shorter survival in early and late stage [26, 31]. We found an *NRAS* mutation in 14% of melanomas, and a single alteration was identified in most cases (62%). *NRAS* mutations are usually mutually exclusive with other oncogenic drivers, such as *BRAF*, *KRAS* and *c-kit* mutations [32, 33]. However, we only observed a coexistence of *NRAS* and *BRAF* mutations in few cases (two samples), consistent with the results from previous studies [29, 31, 34]. No co-mutations were observed between *NRAS* and *KRAS* or *BRAF* with *KRAS* mutations.

Sex biased in cancer driver genes and biomarkers have been previously described [35], and our work is one of the few that studied a possible association between sex-biased mutations in melanoma. Although sex was not a significant factor in our study to predict the presence or absence of an oncogenic alteration, we observed that *NRAS* mutations were predominant in women (*P* = 0.03), whereas *TP53* mutations were more frequent in men (*P* = 0.03). These results provide further evidence on the profit that *BRAF* wt and women patients could gain from NGS testing and the total number of patients (53%) that could benefit of targeted therapies. Less common mutations were only observed in men (*ERBB4*, *STK11*, *FGFR1*, *FGFR3*, *KRAS*, *MET* and *AKT1*). Therefore, these results suggest, as Gupta et al. [36] have previously observed, that there is a gender disparity in the accumulate number of mutations in melanoma with men having a higher and more varied number of somatic mutations than women.

Overall, NGS platforms should be designed and/or selected according to the needs of each institution. There are currently different NGS guidelines providing recommendations to serve as a reference for NGS in house implementation and validation [15, 37]. Minimum samples requirements, such as the presence of at least 20% of tumor cells on FFPE sample is an important criterion to consider, in order to prevent false negative results. Thus, tissue quality control for molecular testing is essential to assess whether the percentage of tumor in a given specimen reaches the detection threshold of the specific molecular test. An important step is to develop an algorithm to select which patients will be assessable for NGS analysis and preserve FFPE samples when necessary for other molecular tests.

At our institution we chose the OST panel to use as the sole platform not only in melanoma but also in other solid tumors, optimizing tumor tissue material, resources and personnel. This approach provides a complete molecular characterization of patients, identifying clinically actionable mutations as well as potential alterations associated with sensitivity to specific drugs.

## Abbreviations

CI: confidence interval
FFPE: formalin-fixed and paraffin embedded
MAPK: mitogen activated protein kinase
NGS: next generation sequencing
OST: oncomine solid tumor
RT-PCR: real-time polymerase chain reaction
wt: wild-type

## Declarations

### Copyright

© The Author(s) 2020.
